# Household wealth index is associated with stunting among children under 5: a cross-sectional analysis of the Lao Social Indicator Survey II

**DOI:** 10.1186/s40101-025-00402-w

**Published:** 2025-07-12

**Authors:** Soulattana Vongsakit, Kumiko Ohara, Yuki Fujita, Akihiro Takada, Katsuyasu Kouda

**Affiliations:** 1 Center of Nutrition, Department of Hygiene and Health Promotion, Ministry of Health, Xiangda Village, Xaysettha District, Vientiane Capital, Lao PDR; 2 Department of Hygiene and Public Health, Faculty of Medicine, Kansai Medical University, 2-5-1 Shin-machi, Hirakata, Osaka 573-1010 Japan; 3 Department of Epidemiology for Community Health and Medicine, Kyoto Prefectural University of Medicine, 465 Kajii-cho, Kawaramachi-Hirokoji, Kamigyo, Kyoto 602-8566 Japan

**Keywords:** Epidemiology, Socioeconomic, Stunting

## Abstract

**Background:**

Stunting in early life is associated with increased morbidity and mortality among children under 5, as well as impaired health and educational and economic performance in later life. However, few studies have investigated risk factors associated with stunting using nationwide representative data in Lao People’s Democratic Republic (Lao PDR). The present study investigated the association of the household wealth index with stunting among children under 5 in Lao PDR using data from the Lao Social Indicator Survey II (LSIS II).

**Methods:**

The present cross-sectional study used secondary data from the LSIS II in 2017. The survey used multi-stage stratified cluster sampling, covering all 18 provinces with 1170 clusters (village), resulting in a sample size of 23,400 households. The final analysis included 11,339 (weighted) children under 5. Multivariable logistic regression analysis was performed to examine associated factors.

**Results:**

Risk factors significantly associated with stunting were no health insurance coverage, minority ethnic groups, having ≥ 7 family members in the household, a poor household wealth index, having ≥ 2 children under 5 in the household, living in rural areas, living in the southern part, and low birth weight. Among them, household wealth index was significantly associated with stunting, independent of other socioeconomic risk factors.

**Conclusions:**

The household wealth index was significantly associated with child stunting. Given the high prevalence of stunting in Lao PDR, there may be a need for the government to implement programs to improve household socioeconomic status in order to address stunting in Lao PDR.

## Introduction

Stunting is defined as a child failing to grow to the proper height for their age, with a height-for-age *Z* score (HAZ) lower than − 2 standard deviations (SD) of the World Health Organization (WHO) Child Growth Standards median [1]. Stunting is associated with increased morbidity and mortality, loss of physical growth potential, reduced neurodevelopmental and cognitive function, and an elevated risk of chronic disease in adulthood [2–4]. There is growing evidence of connections between stunting in early life and impaired health and educational and economic performance in later life [3]. The prevalence of stunting is 22.3% of children under age 5 worldwide, and the number of stunted children under 5 in 2022 was 148.1 million [5]. Risk factors of stunting have been reported to be chronic or recurrent undernutrition, recurrent infections, poor maternal health and nutrition, suboptimal caregiving practices, and inadequate psychosocial stimulation [6], parental and child characteristics, inadequate food intake, access to health services and care, and environmental, household health, and socioeconomic factors [7, 8]. Stunting among children under 5 is most prevalent in South Asia and sub-Saharan Africa than elsewhere [5]. The Lao People’s Democratic Republic (Lao PDR) has one of the highest stunting rates in Southeast Asia; the Lao government prioritizes reducing stunting and improving child health outcomes [9, 10]. Despite recent economic growth, poverty and rural–urban disparities persist, particularly in rural and remote areas where households lack access to essential health services, clean water, sanitation, and adequate nutrition in Lao PDR [11].

Socioeconomic disparities and education level are the main social determinants of stunting [12, 13], though their impact varies by country [14]. Previous studies have indicated that the wealth index serves as a fair proxy for household wealth in the absence of income data [15, 16]. Since the LSIS II did not collect household income data, Therefore, we used the wealth index as a measure of the household’s economic status. The household wealth index is defined as a comprehensive measure of a household’s cumulative living standard. In this study, the household wealth index was constructed using principal component analysis (PCA) based on household assets, dwelling characteristics, water and sanitation, and other characteristics that are related to household’s wealth [17]. We hypothesized that lower household wealth predicts with an increased risk of stunting and that the wealth index provides a stronger and more comprehensive measure of socioeconomic influence compared to individual-level factors. While previous studies on stunting in Laos have mostly been based on regional and community settings and limited studies have reported the association between household wealth index and child stunting using national-level data. Therefore, the present study using nationally representative Lao Social Indicator Survey (LSIS) II data from 2017 to investigate the association between household wealth index and stunting among children under 5.

## Methods

### Data source

The present cross-sectional study used secondary data from the LSIS II in 2017. The LSIS II is a nationally representative multi-purpose household survey conducted by the Lao Statistics Bureau (LSB) in collaboration with the Ministry of Health and Ministry of Education and Sports. The LSIS II followed the sixth global round of the Multiple Indicator Cluster Survey program (MICS6) with technical support from the United Nations Children’s Fund (UNICEF). The LSIS II comprised the following six questionnaires: (1) a household questionnaire, (2) a water quality testing questionnaire, (3) a questionnaire for individual women aged 15–49 years, (4) a questionnaire for individual men aged 15–49 years, (5) a questionnaire for children aged 5–17 years, and (6) a questionnaire for children aged under 5 years. The LSIS II used a multi-stage stratified cluster sampling technique for the selection of the survey sample. At the first stage, clusters (village) were selected. At the second stage, households in each cluster were selected. The number of households selected per cluster was determined to be 20 households. The total sample size was 23,400 households, covering all 18 provinces with 1170 villages consisting of 373 urban, 687 rural with road, and 110 rural without road areas.

### Study participants

The study participants were children aged 0 to 59 months. Data were analyzed from both the household questionnaire (*n* = 23,299) and the questionnaire for children under 5 (*n* = 11,812). These questionnaires were successfully combined to obtain data for all 11,812 children. We also obtained interview data from 11,720 children, with a response rate of 99%. For analysis, all missing data and flagged cases were excluded. Variables with unclear responses (do not know/missing) were also dropped. A total of 352 children with implausible values of HAZ and 41 with unclear responses were excluded from the analysis. The final sample size of children under 5 was 11,327 (unweighted) or 11,339 (weighted).

### Definition of stunting

The outcome variable of this study was stunting among children under 5, defined as HAZ below − 2 SD of the WHO Child Growth Standards median. HAZ below − 6 SD and HAZ above + 6 SD were considered implausible values for anthropometry that were flagged according to the 2006 WHO recommended flagging system [18–20].

### Anthropometric measurement

In the LSIS II, body weight and length/height measurement of children under 5 was performed using an electronic scale and a measuring board as recommended by UNICEF to the nearest 0.1 kg and 0.1 cm, respectively, with the child wearing light clothing and no shoes. For children over 2 years of age, the measurement was performed in a standing position. For children less than 2 years of age, the measurement was performed while lying flat.

### Explanatory variables

To assess associations of stunting with socio-demographic factors, 18 exploratory variables at the individual level and household level were selected from questionnaires (1) and (6) based on previous reports [7, 21, 22]. Individual-level factors included sex, child’s age (in months), diarrhea in the last 2 weeks, fever in the last 2 weeks, cough in the last 2 weeks, and health insurance coverage (Table 1). Household-level factors included sex of the household head, age in years of the household head, ethnicity, education level of the household head, mother’s education level, size of the household, household wealth index, source of drinking water, type of toilet facility, number of children under 5 in the household, area of residence, and region (Table 2).

**Table 1:** Individual characteristics of children aged 0–59 months in Lao PDR

		Overall	*n* (%)	Non-stunted	*n* (%)	Stunted	*n* (%)	*P*-value
		11,339	(100)	7594	(67.0)	3745	(33.0)	
Sex								0.033
	Male	5811	(51.2)	3838	(50.5)	1973	(52.7)	
	Female	5528	(48.8)	3756	(49.5)	1772	(47.3)	
Child’s age in months								< 0.001
	< 6	1079	(9.5)	920	(12.1)	159	(4.2)	
	6–11	1159	(10.2)	948	(12.5)	211	(5.6)	
	12–23	2122	(18.7)	1387	(18.3)	735	(19.6)	
	24–35	2289	(20.2)	1371	(18.1)	917	(24.5)	
	36–47	2461	(21.7)	1497	(19.7)	964	(25.8)	
	48–59	2229	(19.6)	1470	(19.4)	758	(20.3)	
Diarrhea in the last 2 weeks								0.001
	Yes	732	(6.4)	455	(6.0)	277	(7.4)	
	No	10,607	(93.6)	7139	(94.0)	3468	(92.6)	
Fever in the last 2 weeks								0.024
	Yes	1961	(17.3)	1349	(17.8)	611	(16.3)	
	No	9378	(82.7)	6245	(82.2)	3134	(83.7)	
Cough in the last 2 weeks								0.31
	Yes	1704	(15.0)	1150	(15.1)	554	(14.8)	
	No	9635	(85.0)	6444	(84.9)	3191	(85.2)	
Covered by health insurance								< 0.001
	Yes	1548	(13.7)	1237	(16.3)	310	(8.3)	
	No	9791	(86.3)	6357	(83.7)	3435	(91.7)	

*n* number

Values are presented as *n* (%)

*P*-values were calculated using Pearson’s chi-squared test

**Table 2:** Household characteristics of children aged 0–59 months in Lao PDR

		Overall	*n* (%)	Non-stunted	*n* (%)	Stunted	*n* (%)	*P*-value
		11,339	(100)	7594	(67.0)	3745	(33.0)	
Sex of household head								< 0.001
	Male	10,180	(89.8)	6734	(88.7)	3446	(92.0)	
	Female	1159	(10.2)	860	(11.3)	299	(8.0)	
Age in years of household head								< 0.001
	≤ 24	480	(4.2)	283	(3.7)	197	(5.3)	
	25–34	3239	(28.6)	2035	(26.8)	1204	(32.1)	
	35–44	2600	(22.9)	1760	(23.2)	840	(22.4)	
	> 44	5020	(44.3)	3516	(46.3)	1504	(40.2)	
Ethnicity								< 0.001
	Lao-Tai	6396	(56.4)	4913	(64.7)	1483	(39.6)	
	Mon-Khmer	2865	(25.3)	1626	(21.4)	1239	(33.1)	
	Hmong-Mien	1653	(14.6)	823	(10.8)	830	(22.2)	
	Chinese-Tibetan	310	(2.7)	159	(2.1)	151	(4.0)	
	Other	115	(1.0)	73	(1.0)	42	(1.1)	
Education level of household head								< 0.001
	None or early childhood education	1938	(17.1)	1106	(14.6)	832	(22.2)	
	Primary	4982	(44.0)	3241	(42.7)	1741	(46.5)	
	Lower secondary	2148	(18.9)	1490	(19.6)	658	(17.5)	
	Upper secondary	792	(7.0)	602	(7.9)	190	(5.1)	
	Post-secondary/non-tertiary	652	(5.7)	514	(6.8)	138	(3.7)	
	Higher	827	(7.3)	640	(8.4)	186	(5.0)	
Mother’s education level								< 0.001
	None or early childhood education	2469	(21.8)	1363	(17.9)	1106	(29.5)	
	Primary	4476	(39.5)	2929	(38.6)	1547	(41.3)	
	Lower secondary	2306	(20.3)	1621	(21.3)	685	(18.3)	
	Upper secondary	879	(7.7)	686	(9.0)	193	(5.2)	
	Post-secondary/non-tertiary	335	(3.0)	268	(3.6)	67	(1.8)	
	Higher	874	(7.7)	727	(9.6)	147	(3.9)	
Size of household								< 0.001
	≤ 6 members	7278	(64.2)	5066	(66.7)	2212	(59.1)	
	≥ 7 members	4061	(35.8)	2528	(33.3)	1533	(40.9)	
Household wealth index								< 0.001
	Poorest	2904	(25.6)	1511	(19.9)	1393	(37.2)	
	Poorer	2428	(21.4)	1437	(18.9)	991	(26.4)	
	Middle	2135	(18.8)	1492	(19.7)	643	(17.2)	
	Richer	2015	(17.8)	1556	(20.5)	460	(12.3)	
	Richest	1857	(16.4)	1598	(21.0)	258	(6.9)	
Source of drinking water								< 0.001
	Improved	9253	(81.6)	6441	(84.8)	2812	(75.1)	
	Unimproved	2086	(18.4)	1153	(15.2)	933	(24.9)	
Type of toilet facility								< 0.001
	Improved	7753	(68.4)	5595	(73.7)	2158	(57.6)	
	Unimproved	3586	(31.6)	1999	(26.3)	1587	(42.4)	
Number of children under 5 in household								< 0.001
	1	6410	(56.5)	4523	(59.6)	1887	(50.4)	
	≥ 2	4929	(43.5)	3071	(40.4)	1858	(49.6)	
Area of residence								< 0.001
	Urban	3027	(26.7)	2376	(31.3)	651	(17.4)	
	Rural with road	6938	(61.2)	4436	(58.4)	2503	(66.8)	
	Rural without road	1373	(12.1)	782	(10.3)	591	(15.8)	
Region								< 0.001
	North	3552	(31.3)	2173	(28.6)	1380	(36.8)	
	Central	5435	(47.9)	3868	(50.9)	1567	(41.9)	
	South	2351	(20.7)	1553	(20.5)	798	(21.3)	

*n* number

Values are presented as *n* (%)

*P* values were calculated using Pearson's chi-squared test

The main sources of drinking water were divided into two categories (improved sources: piped into dwelling, piped into the compound, yard, or plot, piped to neighbor, public tap, or standpipe, borehole or tubewell, protected well, protected spring, rainwater collection, tanker truck, cart with small tank/drum, bottled water and sachet water; and unimproved sources: unprotected well, unprotected spring, surface water, rivers, reservoirs, lakes, ponds, streams, canals, and irrigation channels).

Toilet facility was divided into two categories (improved: flush/pour, flush to piped sewer systems, flush to septic tanks or pit latrines; pit latrines with slabs and composting toilets (including twin pit latrines with slabs and container-based sanitation) and flush to do not know where; and unimproved: flush/pour flush to open drain, pit latrines without slab/open pit, bucket, hanging toilet/hanging latrine, and no facility/bush/field) [23, 24].

The household wealth index was available in the LSIS II dataset and was divided into five equal parts (quintiles): poorest, poorer, middle, richer, and richest [17, 24]. We used this variable to approximate the socioeconomic position of households. The following assets were used in calculations: main material of dwelling floor, roof, and external walls; possession by the household of a fixed telephone line, radio, clock, sofa/wooden settee, bed/mattress, electricity, television, refrigerator, fan, water pump, air-conditioner, washing machine, CD/DVD player/home theater, iron, rice cooker/steamed cooker, watch, bicycle, motorcycle or scooter, animal-drawn cart, car, truck or van, boat with a motor, tak tak, computer/tablet, mobile phone, internet at home, agricultural land, livestock, herds other than farm animals or poultry, bank account; type of the cookstove, type of fuel or energy source used for the cookstove, and location where the cooking is done; space heating, type of fuel and energy used for space heating; what is used to light the household; source of drinking water; location of water source; reasons for insufficient quantity of water; type and location of sanitation facility, sharing of sanitation facilities; and place for handwashing and availability of soap [17, 24].

### Data for sub-analysis (information for birthweight and mother’s age)

As there was no information regarding birthweight and the mother’s age in (1) the household questionnaire and (6) the questionnaire for children under 5, data from (3) the questionnaire for individual women aged 15–49 years (*n* = 26,088) were used in the sub-analysis, in combination with (1) and (6). All missing data and flagged cases were excluded, and variables with unclear responses were dropped. Completed data for birthweight and the mother’s age were obtained from 3753 children (weighted), as shown in Tables 3 and 4.

**Table 3:** Individual characteristics of children aged 0–59 months included in sub-analysis in Lao PDR

		Overall	*n* (%)	Non-stunted	*n* (%)	Stunted	*n* (%)	*P*-value
		3753	(100)	2804	(74.7)	949	(25.3)	
Sex								0.15
	Male	1959	(52.2)	1438	(51.3)	521	(54.9)	
	Female	1794	(47.8)	1366	(48.7)	428	(45.1)	
Child’s age in months								< 0.001
	< 6	723	(19.3)	620	(22.1)	103	(11.0)	
	6–11	755	(20.1)	639	(22.8)	116	(12.2)	
	12–23	1311	(34.9)	924	(32.9)	387	(40.7)	
	24–35	315	(8.4)	193	(6.9)	122	(12.8)	
	36–47	309	(8.3)	202	(7.2)	107	(11.3)	
	48–59	340	(9.0)	226	(8.1)	114	(12.0)	
Diarrhea in the last 2 weeks								0.50
	Yes	268	(7.1)	207	(7.4)	61	(6.5)	
	No	3485	(92.9)	2597	(92.6)	888	(93.5)	
Fever in the last 2 weeks								0.03
	Yes	654	(17.4)	511	(18.2)	143	(15.1)	
	No	3099	(82.6)	2293	(81.8)	806	(84.9)	
Cough in the last 2 weeks								0.91
	Yes	529	(14.1)	383	(13.7)	146	(15.4)	
	No	3224	(85.9)	2421	(86.3)	803	(84.6)	
Covered by health insurance								< 0.001
	Yes	715	(19.1)	575	(20.5)	141	(14.8)	
	No	3038	(80.9)	2229	(79.5)	808	(85.2)	
Birthweight								< 0.001
	< 2500 g	341	(9.1)	201	(7.2)	140	(14.8)	
	≥ 2500 g	3412	(90.9)	2603	(92.8)	809	(85.2)	

*n* number

Values are presented as *n* (%)

*P*-values were calculated using Pearson's chi-squared test

**Table 4:** Household characteristics of children aged 0–59 months included in sub-analysis in Lao PDR

		Overall	*n* (%)	Non-stunted	*n* (%)	Stunted	*n* (%)	*P*-value
		3753	(100)	2804	(74.7)	949	(25.3)	
Sex of household head								0.001
	Male	3325	(88.6)	2455	(87.6)	869	(91.6)	
	Female	428	(11.4)	349	(12.4)	80	(8.4)	
Age in years of household head								< 0.001
	≤ 24	155	(4.1)	91	(3.2)	64	(6.7)	
	25–34	1011	(27.0)	717	(25.6)	294	(31.0)	
	35–44	765	(20.4)	568	(20.3)	197	(20.8)	
	> 44	1822	(48.5)	1428	(50.9)	394	(41.5)	
Ethnicity								< 0.001
	Lao-Tai	2440	(65.0)	1977	(70.5)	462	(48.7)	
	Mon-Khmer	743	(19.8)	491	(17.5)	253	(26.6)	
	Hmong-Mien	467	(12.5)	262	(9.3)	205	(21.6)	
	Chinese-Tibetan	76	(2.0)	52	(1.9)	24	(2.6)	
	Other	27	(0.7)	22	(0.8)	5	(0.5)	
Education level of household head								0.001
	None or early childhood education	485	(12.9)	324	(11.6)	161	(16.9)	
	Primary	1463	(39.0)	1082	(38.6)	381	(40.1)	
	Lower secondary	778	(20.7)	592	(21.1)	186	(19.6)	
	Upper secondary	309	(8.2)	248	(8.9)	61	(6.4)	
	Post-secondary/non-tertiary	323	(8.6)	254	(9.0)	69	(7.3)	
	Higher	395	(10.5)	304	(10.8)	92	(9.7)	
Mother’s education level								< 0.001
	None or early childhood education	433	(11.5)	286	(10.2)	146	(15.4)	
	Primary	1316	(35.1)	946	(33.8)	370	(39.0)	
	Lower secondary	934	(24.9)	691	(24.6)	243	(25.6)	
	Upper secondary	437	(11.6)	359	(12.8)	78	(8.3)	
	Post-secondary/non-tertiary	156	(4.2)	123	(4.4)	34	(3.5)	
	Higher	477	(12.7)	399	(14.2)	78	(8.2)	
Size of household								0.04
	≤ 6 members	2360	(62.9)	1787	(63.7)	573	(60.4)	
	≥ 7 members	1393	(37.1)	1017	(36.3)	376	(39.6)	
Household wealth index								< 0.001
	Poorest	553	(14.7)	343	(12.2)	210	(22.1)	
	Poorer	696	(18.6)	458	(16.3)	238	(25.1)	
	Middle	766	(20.4)	558	(19.9)	208	(21.9)	
	Richer	837	(22.3)	670	(24.0)	167	(17.6)	
	Richest	901	(24.0)	775	(27.6)	126	(13.3)	
Source of drinking water								< 0.001
	Improved	3276	(87.3)	2499	(89.1)	778	(81.9)	
	Unimproved	477	(12.7)	305	(10.9)	171	(18.1)	
Type of toilet facility								< 0.001
	Improved	3008	(80.2)	2302	(82.1)	706	(74.4)	
	Unimproved	745	(19.8)	502	(17.9)	243	(25.6)	
Number of children under 5 in household								< 0.001
	1	1734	(46.2)	1390	(49.6)	344	(36.3)	
	≥ 2	2019	(53.8)	1414	(50.4)	605	(63.7)	
Area of residence								< 0.001
	Urban	1339	(35.7)	1096	(39.1)	243	(25.6)	
	Rural with road	2104	(56,1)	1516	(54.1)	588	(62.0)	
	Rural without road	310	(8.2)	192	(6.8)	118	(12.4)	
Region								0.04
	North	1216	(32.4)	845	(30.1)	372	(39.2)	
	Central	1931	(51.4)	1512	(53.9)	419	(44.1)	
	South	606	(16.2)	447	(16.0)	158	(16.7)	
Mother’s age in years								0.04
	15–19	303	(8.1)	211	(7.5)	91	(9.6)	
	20–34	3075	(81.9)	2301	(82.1)	774	(81.5)	
	35–49	375	(10.0)	292	(10.4)	84	(8.9)	

*n* number

*P*-values were calculated using Pearson’s chi-squared test

Values are presented as *n* (%)

### Statistical analysis

Data analysis was performed using STATA software, version IC 16.1 (Copyright 1985–2019 Statacorp LLC, 4905 Lakeway Drive, College Station, TX 77845, USA) and SPSS Statistics Desktop for Japan, Version 26 (IBM Japan, Ltd., Tokyo, Japan). Sampling weights in each cluster were used in all analyses to adjust for the unequal number of children in each cluster to make the estimates representative of the population level [25]. Sampling weights were obtained from the LSIS II dataset. Pearson’s chi-squared test was used to compare the proportion of stunting and that of non-stunting for each variable. Univariate logistic regression analysis was used to determine variables associated with stunting among children. Variables with *P* < 0.20 in the univariable analysis were included in the subsequent multivariable logistic regression analysis. Multicollinearity among the independent variables was checked using the variance inflation factor (VIF), with a VIF > 5 indicating the presence of multicollinearity. *P* value < 0.05 was considered statistically significant.

We also calculated the adjusted mean value of HAZ in each household wealth index category using a linear model with the household wealth index and potential confounding factors. Multivariable linear regression analysis was performed to assess relationships between HAZ and household wealth index categories, and trends from the lowest to the highest quintile were evaluated. The dependent variable was HAZ in each subject, and independent variables were household wealth index categories and potential confounding factors. A *P*-value less than 0.05 was considered statistically significant.

We also performed PCA, which was conducted on 20 variables to reduce variables. Variables included in the PCA were numeric or ordinal categorical variables that were converted appropriately for analysis. The number of components to retain was determined based on the Kaiser criterion (Eigenvalues > 1) and the scree plot. After identifying key principal components (PCs), component scores were predicted and used as predictor variables in the multivariable logistic regression model to identify factors associated with stunting.

## Results

Table 1 shows the individual characteristics of children aged 0–59 months. There were 33% of stunted children in Laos. Approximately 85% of children were not covered by health insurance. Table 2 shows the household characteristics of children aged 0–59 months. The majority of household heads were male, about half of household heads were aged older than 44 years, and one in five household heads and mothers had no education or early childhood education. More than half of children were of Lao-Tai ethnicity, lived in rural areas with roads, and lived in the central part. One in four children lived in the poorest households, and 35% of children lived with ≥ 7 household members. Approximately 45% of households had ≥ 2 children under age 5, and about 20% of households had an unimproved source of drinking water. One in three households had an unimproved toilet facility.

Tables 1 and 2 also show differences in characteristics between non-stunted and stunted children aged 0–59 months. The overall prevalence of stunting among all children was 33%. There were significant differences between non-stunted and stunted children in characteristics of sex, age, diarrhea, fever, health insurance coverage, sex of the household head, age of the household head, ethnicity, education level of the household head and mother, size of the household, household wealth index, source of drinking water, toilet facility, number of children under 5 in the household, area of residence (urban and rural), and region (north, central, and south).

Table 3 shows individual characteristics of children aged 0–59 months included in the sub-analysis. Approximately 9% of children were born with low birthweight < 2500 g. Table 4 shows the household characteristics of children aged 0–59 months included in the sub-analysis. The majority of mothers were aged between 20 and 34 years; about 8% and 10% of mothers were aged 15–19 and 35–49 years, respectively.

Tables 3 and 2 also show differences in characteristics between non-stunted and stunted children aged 0–59 months included in the sub-analysis. There were significant differences between non-stunted and stunted children in terms of birthweight and the mother’s age. There were also significant differences between non-stunted and stunted children in age, fever, health insurance coverage, sex of the household head, age of the household head, ethnicity, education level of the household head and mother, size of the household, household wealth index, source of drinking water, toilet facility, number of children under 5 in the household, area of residence (urban and rural), and region (north, central, and south).

Table 5 shows associations between various factors and stunting among children aged 0–59 months. Multivariable logistic regression analysis revealed that children who are male, of older age (6–59 months), not covered by health insurance, of minority ethnic groups (Mon-Khmer, Hmong-Mien, Chinese-Tibetan), with ≥ 7 family members in the household, with a poor household wealth index, with ≥ 2 children under 5 in the household, living in rural areas, and living in the southern part were more likely to be stunted. On the other hand, children with the household head having upper secondary education were less likely to be stunted. Household wealth index was associated with stunting independent of other factors.

**Table 5:** Crude and adjusted odds ratios for stunting in children under 5 (*n* = 11,339) in Lao PDR

		Univariable analysis			Multivariable analysis			
		cOR	[95% CI]	*P*-value	aOR	[95% CI]	*P*-value	VIF
Sex (Ref, Female)								1.00
	Male	1.09	(1.00–1.18)	0.03	1.11	(1.02–1.21)	0.02	
Child’s age in months (Ref, < 6)								1.02
	6–11	1.28	(1.02–1.61)	0.03	1.36	(1.08–1.72)	0.01	
	12–23	3.06	(2.53–3.70)	< 0.01	3.58	(2.94–4.36)	< 0.01	
	24–35	3.86	(3.20–4.66)	< 0.01	4.55	(3.74–5.53)	< 0.01	
	36–47	3.72	(3.08–4.48)	< 0.01	4.38	(3.61–5.32)	< 0.01	
	48–59	2.98	(2.46–3.60)	< 0.01	3.47	(2.85–4.23)	< 0.01	
Diarrhea in the last 2 weeks (Ref, No)								1.05
	Yes	1.25	(1.07–1.46)	< 0.01	1.15	(0.97–1.37)	0.1	
Fever in the last 2 weeks (Ref, No)								1.05
	Yes	0.90	(0.81–1.00)	0.06	0.95	(0.85–1.07)	0.40	
Cough in the last 2 weeks (Ref, No)								
	Yes	0.97	(0.87–1.08)	0.61				
Covered by health insurance (Ref, Yes)								1.36
	No	2.15	(1.89–2.46)	< 0.01	1.23	(1.05–1.45)	0.01	
Sex of household head (Ref, Female)								1.10
	Male	1.47	(1.28–1.69)	< 0.01	1.03	(0.88–1.20)	0.74	
Age in years of household head (Ref, > 44)								1.38
	≤ 24	1.63	(1.34–1.97)	< 0.01	0.97	(0.78–1.21)	0.78	
	25–34	1.38	(1.26–1.52)	< 0.01	1.06	(0.94–1.19)	0.36	
	35–44	1.12	(1.01–1.24)	0.04	0.99	(0.89–1.12)	0.98	
Ethnicity (Ref, Lao-Tai)								1.26
	Mon-Khmer	2.52	(2.30–2.77)	< 0.01	1.51	(1.34–1.69)	< 0.01	
	Hmong-Mien	3.34	(2.98–3.74)	< 0.01	2.21	(1.92–2.55)	< 0.01	
	Chinese-Tibetan	3.13	(2.48–3.94)	< 0.01	2.04	(1.56–2.66)	< 0.01	
	Other	1.92	(1.31–2.82)	< 0.01	1.45	(0.97–2.18)	0.07	
Education level of household head (Ref, Higher)								1.78
	None or early childhood education	2.58	(2.14–3.11)	< 0.01	0.97	(0.76–1.25)	0.84	
	Primary	1.84	(1.55–2.19)	< 0.01	0.85	(0.68–1.06)	0.15	
	Lower secondary	1.52	(1.26–1.83)	< 0.01	0.83	(0.66–1.04)	0.11	
	Upper secondary	1.08	(0.86–1.36)	0.50	0.75	(0.58–0.97)	0.03	
	Post-secondary/non-tertiary	0.92	(0.72–1.18)	0.52	0.84	(0.64–1.10)	0.2	
Mother’s education level (Ref, Higher)								2.01
	None or early childhood education	4.02	(3.31–4.88)	< 0.01	0.90	(0.69–1.17)	0.45	
	Primary	2.62	(2.17–3.16)	< 0.01	0.97	(0.76–1.24)	0.82	
	Lower secondary	2.09	(1.72–2.56)	< 0.01	1.05	(0.82–1.33)	0.71	
	Upper secondary	1.40	(1.10–1.77)	< 0.01	1.03	(0.79–1.35)	0.82	
	Post-secondary/non-tertiary	1.24	(0.90–1.70)	0.2	1.04	(0.74–1.46)	0.81	
Size of household (Ref, ≤ 6 members)								1.32
	≥ 7 members	1.39	(1.28–1.51)	< 0.01	1.17	(1.06–1.30)	< 0.01	
Household wealth index (Ref, Richest)								3.02
	Poorest	5.71	(4.91–6.64)	< 0.01	3.05	(2.41–3.87)	< 0.01	
	Poorer	4.27	(3.66–4.98)	< 0.01	2.72	(2.21–3.35)	< 0.01	
	Middle	2.67	(2.27–3.14)	< 0.01	1.99	(1.65–2.42)	< 0.01	
	Richer	1.83	(1.55–2.16)	< 0.01	1.63	(1.36–1.96)	< 0.01	
Source of drinking water (Ref, Improved)								1.22
	Unimproved	1.85	(1.68–2.04)	< 0.01	1.07	(0.96–1.20)	0.21	
Type of toilet facility (Ref, Improved)								1.71
	Unimproved	2.06	(1.89–2.23)	< 0.01	1.06	(0.94–1.19)	0.34	
Number of children under 5 (Ref, 1)								1.17
	≥ 2	1.45	(1.34–1.57)	< 0.01	1.12	(1.02–1.23)	0.02	
Area of residence (Ref, Urban)								1.48
	Rural with road	2.06	(1.86–2.28)	< 0.01	1.16	(1.03–1.32)	0.02	
	Rural without road	2.76	(2.41–3.17)	< 0.01	1.22	(1.02–1.44)	0.03	
Region (Ref, Central)								1.11
	North	1.57	(1.43–1.71)	< 0.01	1.05	(0.95–1.17)	0.33	
	South	1.27	(1.14–1.41)	< 0.01	1.16	(1.03–1.30)	0.01	

*cOR* crude odds ratio, *aOR* adjusted odds ratio, *Ref* reference, *VIF* variance inflation factor

Multivariable logistic regression analysis was performed with independent variables including sex, children’s age in months, diarrhea in the last 2 weeks, fever in the last 2 weeks, health insurance coverage, sex of the household head, age in years of the household head, ethnicity, education level of the household head, mother’s education level, size of the household, household wealth index, source of drinking water, type of toilet facility, number of children under 5 in the household, area of residence, and region

Table 6 shows associations between various factors and stunting among children aged 0–59 months included in the sub-analysis. Multivariable logistic regression analysis revealed that children with low birthweight < 2500 g, male sex, older age (12–59 months), fever in the last 2 weeks, household head aged ≤ 24 years, minority ethnic group status (Mon-Khmer, Hmong-Mien), a poor household wealth index, ≥ 2 children under 5 in the household, and residence in rural areas were more likely to be stunted. On the other hand, children with the household head having lower and upper secondary education were less likely to be stunted. Household wealth index was associated with stunting independent of other factors.

**Table 6:** Crude and adjusted odds ratios for stunting in children under 5 (*n *= 3753) in Lao PDR

		Univariable analysis			Multivariable analysis			
		cOR	[95% CI]	*P*-value	aOR	[95% CI]	*P*-value	VIF
Sex (Ref, Female)								1.01
	Male	1.16	(0.99–1.34)	0.05	1.21	(1.03–1.42)	0.02	
Child's age in months (Ref, < 6)								1.20
	6–11	1.09	(0.82–1.45)	0.6	1.14	(0.84–1.54)	0.39	
	12–23	2.51	(1.97–3.19)	< 0.01	2.91	(2.25–3.75)	< 0.01	
	24–35	3.78	(2.78–5.14)	< 0.01	3.39	(2.44–4.72)	< 0.01	
	36–47	3.19	(2.33–4.36)	< 0.01	2.88	(2.04–4.07)	< 0.01	
	48–59	3.01	(2.22–4.09)	< 0.01	2.88	(2.06–4.05)	< 0.01	
Diarrhea in the last 2 weeks (Ref, No)								
	Yes	0.87	(0.65–1.17)	0.35				
Fever in the last 2 weeks (Ref, No)								1.24
	Yes	0.79	(0.65–0.97)	0.03	0.78	(0.61–0.99)	0.04	
Cough in the last 2 weeks (Ref, No)								1.24
	Yes	1.15	(0.94–1.14)	0.18	1.21	(0.95–1.56)	0.13	
Covered by health insurance (Ref, Yes)								1.34
	No	1.48	(1.21–1.81)	< 0.01	1.15	(0.89–1.49)	0.29	
Sex of household head (Ref, Female)								1.11
	Male	1.54	(1.19–1.99)	< 0.01	1.13	(0.84–1.50)	0.42	
Age in years of household head (Ref, > 44)								1.45
	≤ 24	2.54	(1.81–3.56)	< 0.01	1.75	(1.17–2.62)	< 0.01	
	25–34	1.49	(1.25–1.77)	< 0.01	1.26	(0.99–1.58)	0.05	
	35–44	1.26	(1.03–1.53)	0.02	1.17	(0.92–1.48)	0.20	
Ethnicity (Ref, Lao-Tai)								1.22
	Mon-Khmer	2.20	(1.83–2.64)	< 0.01	1.32	(1.07–1.71)	0.02	
	Hmong-Mien	3.35	(2.72–4.13)	< 0.01	2.44	(1.89–3.18)	< 0.01	
	Chinese-Tibetan	2.01	(1.23–3.29)	< 0.01	1.41	(0.82–2.50)	0.23	
	Other	0.98	(0.37–2.61)	0.97	1.24	(0.44–3.40)	0.68	
Education level of household head (Ref, Higher)								1.74
	None or early childhood education	1.64	(1.22–2.22)	< 0.01	1.10	(0.73–1.66)	0.66	
	Primary	1.16	(0.90–1.51)	0.25	0.76	(0.53–1.08)	0.12	
	Lower secondary	1.04	(0.78–1.38)	0.80	0.69	(0.48–0.98)	0.04	
	Upper secondary	0.81	(0.56–1.17)	0.26	0.64	(0.42–0.98)	0.04	
	Post-secondary/non-tertiary	0.90	(0.63–1.29)	0.57	0.98	(0.66–1.45)	0.92	
Mother’s education level (Ref, Higher)								1.91
	None or early childhood education	2.62	(1.91–3.59)	< 0.01	0.70	(0.45–1.08)	0.11	
	Primary	2.01	(1.53–2.63)	< 0.01	0.9	(0.62–1.31)	0.58	
	Lower secondary	1.80	(1.36–2.40)	< 0.01	1.05	(0.73–1.50)	0.8	
	Upper secondary	1.12	(0.79–1.58)	0.52	0.96	(0.64–1.43)	0.84	
	Post-secondary/non-tertiary	1.41	(0.90–2.22)	0.14	1.03	(0.63–1.69)	0.91	
Size of household (Ref, ≤ 6 members)								1.36
	≥ 7 members	1.15	(0.99–1.34)	0.07	1.03	(0.85–1.26)	0.75	
Household wealth index (Ref, Richest)								2.66
	Poorest	3.74	(2.90–4.83)	< 0.01	1.97	(1.30–2.98)	< 0.01	
	Poorer	3.18	(2.49–4.06)	< 0.01	2.06	(1.46–2.91)	< 0.01	
	Middle	2.28	(1.79–2.92)	< 0.01	1.62	(1.19–2.21)	< 0.01	
	Richer	1.52	(1.18–1.96)	< 0.01	1.37	(1.03–1.81)	0.03	
Source of drinking water (Ref, Improved)								1.25
	Unimproved	1.81	(1.47–2.22)	< 0.01	1.17	(0.92–1.49)	0.21	
Type of toilet facility (Ref, Improved)								1.59
	Unimproved	1.58	(1.33–1.88)	< 0.01	0.86	(0.68–1.10)	0.24	
Number of children under 5 (Ref, 1)								1.30
	≥ 2	1.73	(1.48–2.01)	< 0.01	1.27	(1.04–1.54)	0.02	
Area of residence (Ref, Urban)								1.46
	Rural with road	1.75	(1.48–2.07)	< 0.01	1.39	(1.13–1.71)	< 0.01	
	Rural without road	2.77	(2.12–3.62)	< 0.01	1.89	(1.35–2.63)	< 0.01	
Region (Ref, Central)								1.13
	North	1.59	(1.35–1.87)	< 0.01	1.17	(0.96–1.42)	0.12	
	South	1.28	(1.04–1.58)	0.02	1.36	(1.07–1.72)	0.01	
Birthweight (Ref, ≥ 2500 g)								1.02
	< 2500 g	2.25	(1.79–2.83)	< 0.01	2.35	(1.83–3.02)	< 0.01	
Mother's age in years (Ref, 20–34)								
	15–19	1.29	(0.99–1.67)	0.06	1.16	(0.86–1.56)	0.34	
	35–49	0.86	(0.66–1.11)	0.24	1.09	(0.81–1.46)	0.56	

*cOR* crude odds ratio, *aOR* adjusted odds ratio, *Ref* reference, *VIF* variance inflation factor

Multivariable logistic regression analysis was performed with independent variables including sex, children’s age in months, fever in the last 2 weeks, cough in the last 2 weeks, health insurance coverage, sex of the household head, age in years of the household head, ethnicity, education level of the household head, mother’s education level, size of the household, household wealth index, source of drinking water, type of toilet facility, number of children under 5 in the household, area of residence, region, birthweight, and mother’s age in years

Table 7 presents the results of the PCA using the variables listed in Table 6. We obtain 6 components using the analysis. The first component showed the highest proportion in the analysis. The factor loading from the PCA revealed that component 1 primarily captured socioeconomic characteristics, including wealth index, maternal and household head’s education, toilet facility, source of drinking water, coverage by health insurance, area of residence, and ethnicity. Component 2 represented household structure, particularly the age of the household head and household size. Component 3 related to child health, including fever, cough, and diarrhea. Component 4 reflected household and child demographics such as child’s age, number of children under 5, and household size. Component 5 was dominated by maternal age and region. While component 6 was strongly influenced by sex, birthweight, and region, representing biological characteristics of the child.

**Table 7:** Results of principal component analysis using variable included in Table 6

		Component 1	Component 2	Component 3	Component 4	Component 5	Component 6
	Eigenvalue	3.35	1.67	1.58	1.52	1.16	1.09
	Difference	1.68	0.10	0.05	0.36	0.07	0.09
	Proportion (%)	16.8	8.4	7.9	7.6	5.8	5.4
	Cumulative (%)	16.8	25.1	33.0	40.6	46.4	51.8
Variable		Component score					
	Child’s age in months	− 0.03	− 0.27	− 0.09	0.48	0.30	− 0.01
	Number of children under 5	− 0.10	− 0.15	− 0.20	0.59	0.10	0.04
	Size of household	− 0.07	0.40	− 0.12	0.47	− 0.22	0.00
	Age in years of household head	0.10	0.59	− 0.12	0.15	− 0.13	− 0.04
	Household wealth index	0.46	0.13	− 0.03	0.04	0.04	0.03
	Mother’s education level	0.41	− 0.08	− 0.02	0.05	− 0.25	− 0.01
	Education level of household head	0.32	− 0.42	0.05	0.05	− 0.17	− 0.01
	Type of toilet facility	0.31	0.17	− 0.06	0.04	0.02	− 0.07
	Source of drinking water	0.25	0.07	0.00	−0.03	0.21	− 0.19
	Sex	− 0.01	− 0.03	0.01	− 0.01	− 0.18	0.63
	Cough in the last 2 weeks	0.02	0.05	0.56	0.21	0.01	− 0.06
	Fever in the last 2 weeks	0.03	0.09	0.62	0.15	0.08	0.04
	Diarrhea in the last 2 weeks	− 0.02	0.04	0.45	0.04	− 0.10	0.11
	Covered by health insurance	0.27	− 0.23	0.01	0.12	− 0.27	− 0.08
	Ethnicity	0.32	0.10	− 0.02	− 0.21	0.25	0.11
	Region	0.12	0.04	0.00	0.13	0.37	0.44
	Sex of household head	− 0.07	− 0.26	0.03	0.08	− 0.32	− 0.15
	Area of residence	0.34	0.01	− 0.01	0.16	− 0.06	− 0.08
	Birthweight	0.07	− 0.07	− 0.09	− 0.02	− 0.13	0.54
	Mother’s age in years	0.08	− 0.11	0.01	0.02	0.51	− 0.09

Table 8 presents the association between principal component and stunting among children under 5. Component 1 shows a statistically significant protective effect (OR = 0.76, *P* < 0.001) indicating that children from household with higher socioeconomic status, better maternal and household’s head education, urban residence and improved WASH access were less likely to be stunted. Similarly, component 2 was also significantly associated with lower odds of stunting, representing that household structure was the significantly protective factor. In contrast, component 4 was significantly associated with an increased risk of stunting, suggesting that children from household with multiple and older children were more likely to be stunting. Additionally, component 6 showed a significant protective effect, indicating that children with higher birthweight and female sex were significantly less likely to be stunted.

**Table 8:** Multivariable logistic regression analysis for stunting among children aged 0–59 months using component score in Table 7

PCA factors	Odds ratio	[95% CI]	*P*-value
Component 1: Socioeconomic status	0.76	(0.73–0.80)	< 0.01
Component 2: Household structure	0.83	(0.78–0.88)	< 0.01
Component 3: Child health	0.96	(0.90–1.02)	0.22
Component 4: Household and child demographics	1.26	(1.19–1.34)	< 0.01
Component 5: Maternal age	0.99	(0.93–1.07)	0.86
Component 6: Child’s biological	0.86	(0.80–0.92)	< 0.01

*PCA* principal component analysis, *CI* confidence interval

Dependent variable was stunting. Independent variables were components 1 to 6

**Fig. 1:**
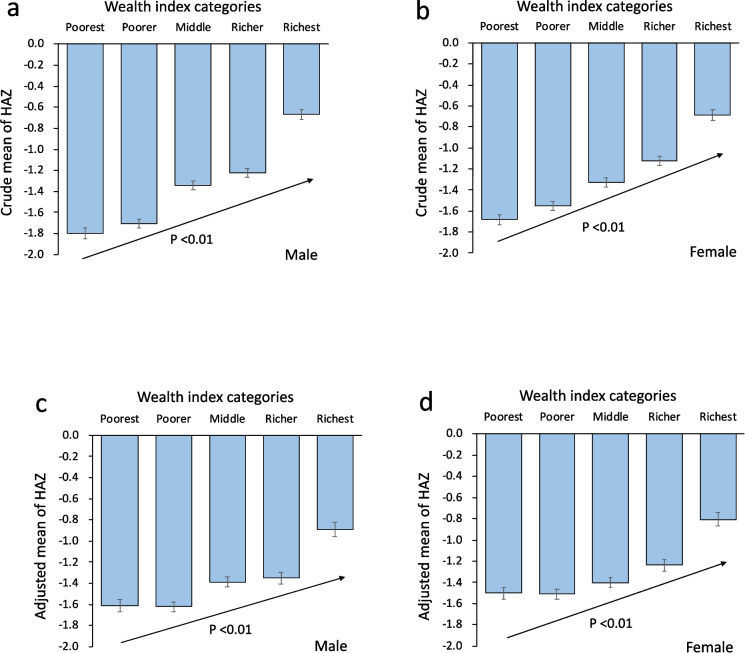
Means and standard errors of HAZ stratified by household wealth index

Figure 1 presents values of HAZ according to household wealth index categories classified by sex. Both crude and adjusted means of HAZ in all household wealth index categories were less than zero. Even children in the richest category had negative values of HAZ level. From the poorest to richest quintile, HAZ increased with increasing household wealth index in both sexes.

## Discussion

This study is the first to clarify the association between stunting and the household wealth index among children under 5 in Lao PDR using data from the LSIS II, a nationally representative sample survey. The findings of the present study highlight the importance of categorizing households according to the household wealth index. Generally, the household wealth index is used to assess household socio-economic status (SES) and is calculated based on household ownership of assets, such as consumer goods, dwelling characteristics, water and sanitation, and other characteristics that are related to the household’s wealth. In the present study, there was a high prevalence of stunting children, and the household wealth index, sex, children’s age, health insurance coverage, ethnicity, size of the household, number of children under 5 in the household, area of residence, region, and birthweight were significantly associated with stunting. Among these risk factors, the household wealth index was a significant predictor of stunting in children under 5, independent of other risk factors (i.e., sex, children’s age, health insurance coverage, ethnicity, size of the household, number of children under 5 in the household, area of residence, region, and birthweight). In addition, the association with stunting appeared to be stronger with the household wealth index than with other risk factors. These findings indicate that children with a lower household SES tend to have a shorter stature. Furthermore, to summarize and capture the essence of information from multivariate data, principal component analysis was conducted; six components were extracted. Component 1 was predominantly composed of socioeconomic variables, including the household wealth index, indicating that better socioeconomic conditions offer a protective effect for stunting. These findings may indicate that socioeconomic conditions, particularly the household wealth index, are one of the factors which are related to stunting.

A previous study conducted in Indonesian children aged 0.5 to 12 years reported that stunting was highly prevalent among children in low SES households [26]. Another study in West Africa found that children living in wealthy households had a lower prevalence of stunting [27]. These findings are consistent with our findings based on the Lao national survey. One potential explanation for this association of stunting with a lower household SES is that poor households may experience food insecurity, leading to low household food diversity and poor feeding practices [28]. In addition, disadvantaged households may be unable to secure healthy meals due to poverty [29]. Indeed, poverty increases the likelihood of individuals being exposed to health-related risks [30, 31]. In contrast, rich households have greater purchasing power for food and other consumer goods needed to ensure children’s health [32]. The findings of the present study highlight the importance of categorizing households according to the household wealth index, ethnicity, education level of the household head, and area of residence in addressing child stunting, as well as the need for policymakers to understand the relationship between stunting and household SES. On the other hand, it has been reported that the Social-Economic-Political-Emotional (SEPE) framework, which includes social and political determinants, as well as emotional stressors, also influences growth trajectories. Children from lower SEPE-status households experience not only material deprivation, such as food insecurity and poor nutrition, but also reduced access to community resources, all of which contribute to growth faltering [33]. SEPE theory suggests that economic status alone does not fully explain these disparities. Children from wealthier households benefit not only from better nutrition but also from greater social capital, political influence, and emotional security, which together enhance growth outcomes [33].

The strength of the present study lies in the fact that this was a large-scale population-based study, with participants recruited by multi-stage stratified cluster sampling. Probability sampling offered an ideal setting to carry out an unbiased evaluation of the relationship between the household wealth index and stunting. Moreover, the large-scale population sample provided sufficient statistical power to adjust for confounding factors. Furthermore, the large sample size helped reduce random sampling errors. Since the analyzed population accounted for 95.9% of the source population, the findings of the present study can be generalized to all children aged 0–59 months in Lao PDR.

This study also has several limitations worth noting, despite the use of representative data. First, due to the cross-sectional study design, the temporal sequence of risk factors and outcomes to be assessed could not be clarified. Second, this study was based on secondary data of the LSIS II in 2017 and thus did not include all potential exposure variables. Finally, since the data used were from 7 years ago, some contexts may have changed, and the reality of the present circumstances may not be fully reflected.

## Conclusion

The prevalence of stunting was found to be high in Lao PDR, and the household wealth index was significantly associated with stunting among children under 5, indicating that children from poorer households are more likely to experience stunting than those from wealthier households. Various other factors were also significantly associated with stunting, including sex, children’s age, health insurance coverage, ethnicity, size of the household, number of children under 5 in the household, area of residence, region, and birth weight. We also found that the household wealth index had the highest contribution to and was a key determinant of stunting. This suggests that wealth disparities play a more prominent role in stunting than other individual or community-level factors. To address the problem of stunting in Lao PDR, there may be a need for the government to implement programs focused on improving household SES and SEPE.

## Data Availability

The data that support the findings of the current paper are available from the corresponding author upon reasonable request.

## Abbreviations

HAZ: Height for age *Z*-score
Lao PDR: Lao People’s Democratic Republic
LSB: Lao Statistics Bureau
LSIS II: Lao Social Indicator Survey II
LBW: Low birthweight
MICS 6: Multiple Indicator Cluster Survey 6
PCA: Principal component analysis
SD: Standard deviation
SES: Socio-economic status
SEPE: Social-Economic-Political-Emotional
UNICEF: United Nations Children’s Fund
WHO: World Health Organization
